# Matrine Ameliorates Colorectal Cancer in Rats via Inhibition of HMGB1 Signaling and Downregulation of IL-6, TNF-*α*, and HMGB1

**DOI:** 10.1155/2018/5408324

**Published:** 2018-01-10

**Authors:** Huizhen Fan, Chunyan Jiang, Baoyuan Zhong, Jianwen Sheng, Ting Chen, Qingqing Chen, Jingtao Li, Hongchuan Zhao

**Affiliations:** ^1^Department of Gastroenterology, The People's Hospital of Yichun City, Yichun, China; ^2^Department of Dermatology, Beijing Hospital of Traditional Chinese Medicine, Beijing, China; ^3^Department of General Surgery, First Affiliated Hospital of Gannan Medical College, Ganzhou, China; ^4^Department of Gastroenterology, China-Japan Friendship Hospital, Beijing, China

## Abstract

*Matrine* may be protective against colorectal cancer (CRC), but how it may work is unclear. Thus, we explored the underlying mechanisms of matrine in CRC. Matrine-related proteins and CRC-related genes and therapeutic targets of matrine in CRC were predicted using a network pharmacology approach. Five targets, including interleukin 6 (IL-6), the 26S proteasome, tumor necrosis factor alpha (TNF-*α*), transforming growth factor beta 1 (TGF-*β*1) and p53, and corresponding high-mobility group box 1 (HMGB1) signaling and T helper cell differentiation were thought to be associated with matrine's mechanism. Expression of predicted serum targets were verified in a 1,2-dimethylhydrazine dihydrochloride-induced CRC model rats that were treated with matrine (ip) for 18 weeks. Data show that matrine suppressed CRC growth and decreased previously elevated expression of IL-6, TNF-*α*, p53, and HMGB1. Matrine may have had a therapeutic effect on CRC via inhibition of HMGB1 signaling, and this occurred through downregulation of IL-6, TNF-*α*, and HMGB1.

## 1. Introduction

Colorectal cancer (CRC) is a malignant colon or rectal tumor and the third most common type of cancer, accounting for ~10% of all cancer cases and ~715,000 premature deaths annually [1, 2]. Conservative drug treatment, directed toward improving the quality of life and symptoms, is an important component of CRC therapy. Chinese herbal medicine has been used as an adjuvant treatment for CRC, but few reports describe mechanistic data for these compounds [3–5]. Matrine is a bioactive component extracted from *Sophora flavescens*, and a few studies have suggested that it may have anticancer activity and may be used as an adjuvant treatment for CRC [6–8]. How matrine exerts an effect, however, is not clear.

Network pharmacology uses systems pharmacology to help researchers to understand drug mechanisms of action [9]. Network pharmacology approaches have been used for drug discovery and design, and the development of biomarkers for disease detection [9, 10]. In Chinese medicine, the approach has been used to elucidate mechanisms of bioactive components of some Chinese herbs [11, 12]. Thus, we suggest that we can understand how matrine effects CRC using a predicted component-target network [13].

Using predicted targets of matrine for treating CRC and a network pharmacology approach, we treated 1,2-dimethylhydrazine dihydrochloride- (DMH-) induced CRC rats with matrine and measured target expression in serum to better understand how matrine may be applied clinically.

## 2. Materials and Methods

### 2.1. Matrine-Related Proteins

PubChem (http://pubchem.ncbi.nlm.nih.gov/) was searched for matrine-related proteins (until November 16, 2015). Because the proteins could be cross-referenced to other National Center for Biotechnology Information (NCBI) databases, the proteins that were tested in bioassays were collected [12]. Nineteen matrine-related proteins were included in the study (Supplementary Table 1).

### 2.2. CRC-Related Genes

CRC-related genes were searched in the NCBI Gene database (http://www.ncbi.nlm.nih.gov/gene) using the key words “colorectal cancer” (until November 16, 2015) [12]. Thirty-five CRC-related genes were included in the study (Supplementary Table 2).

### 2.3. Network Pharmacological Analysis Using Ingenuity Pathways Analysis

CRC-related genes and matrine-related proteins were uploaded into the Ingenuity Pathways Analysis Platform (IPA, http://www.ingenuity.com), which enabled the discovery, visualization, and exploration of molecular interactions to identify the biological mechanisms, pathways, and functions most relevant to genes or proteins of interest. The “core analysis” platform in the IPA was used to assess the uploaded genes and proteins. Scores were negative base 10 logarithms of Fisher's exact test *p* value in the pathway analysis. Significance values for biological functions were assigned to each network by determining *p* values for gene enrichment in the network by comparing these data with the Ingenuity Pathway Knowledge Base [14].

### 2.4. Matrine Solution and Reagents

The matrine ampule (5 ml, 80 mg) was purchased from Baiyunshan Pharmaceutical Co. Ltd. (Guangzhou, China; batch number 20151203), and it was diluted with 10% glucose solution. DMH was purchased from Puzhen Biological Technology Co. Ltd. (Shanghai, China; CAS 306-37-6). ELISA kits used in this study included rat interleukin 6 (IL-6), tumor necrosis factor alpha (TNF-*α*), high-mobility group box 1 (HMGB1) and transforming growth factor beta 1 (TGF-*β*1) ELISA kits (Enzyme-Linked Biotechnology Co. Ltd., Shanghai, China), a rat 26S proteasome ELISA kit (Bio-Medical Assay Co. Ltd., Beijing, China), and a rat tumor protein p53 (p53) ELISA kit (BD Biosciences, CA).

### 2.5. Experimental Rats, Modeling, and Grouping

A total of 32 male Wistar rats (80–100 g; license number SCXK 2016-007) were obtained from the Experimental Animal Center of the Beijing Capital University of Medical Sciences (China). All rats were housed in a temperature-, humidity-, and light-controlled environment, and food and tap water were provided ad libitum. The light-dark cycle was 12 hours (light phase from 06:00 to 18:00). All rats were acclimated in their cages for seven days prior to any experiments. The rodent license for the laboratory (number SYXK 11-00-0039) was issued by the Science and Technology Ministry of China. A colorectal carcinogenesis model was induced in rats using DMH once per week (30 mg/kg, sc) for 18 weeks [15]. Four experimental groups (*n* = 8/group) were established as follows: healthy controls; CRC model controls; and CRC model rats given low (LM) or high (HM) doses of matrine. All animal experimentation was performed under the Prevention of Cruelty to Animals Act (1986) of China and the NIH Guidelines for the Care and Use of Laboratory Animals (USA).

### 2.6. Treatment Schedule

Matrine doses used in the rats were equivalent to clinically relevant human adult doses based on an established formula for human-rat drug conversion. After all rats were acclimatized and grouped, the LM and HM groups received 15 and 30 mg/kg (ip) injections of matrine solution, every three days for 18 weeks. Meanwhile, all rats except for controls underwent colorectal carcinogenesis induction with DMH. Rats were observed daily, and at the end of the experiment, rats were sacrificed and their peripheral blood and colons were collected for analysis.

### 2.7. ELISA

To determine whether matrine administration affected expression of predicted target proteins (IL-6, 26S proteasome, TNF-*α*, TGF-*β*1, p53, and HMGB1) in the serum, protein expression was measured using commercial rat ELISA kits according to the manufacturer's instructions.

### 2.8. Statistical Analysis

Experimental results were expressed as the means ± SD. Statistical differences were analyzed by one-way ANOVA using SPSS Statistics 23. All statistical tests and corresponding *p* values were two sided, and *p* < 0.05 was considered statistically significant.

## 3. Results

### 3.1. Diseases or Disorders Associated with Matrine-Related Proteins

**Figure 1:**
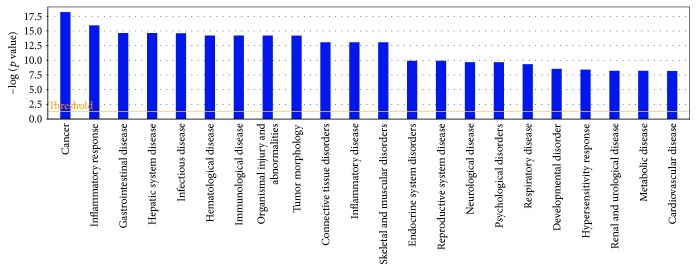
Diseases or disorders associated with matrine-related proteins. Statistical significance gradually decreases from left to right.

Based on IPA analysis, matrine-related proteins were significantly associated with 22 diseases or disorders (Figure 1). The top five diseases or disorders, in order, were cancer, inflammatory responses, gastrointestinal disease, hepatic system disease, and infectious diseases. These data suggest that matrine may be a candidate treatment for cancer.

### 3.2. Networks Associated with Matrine-Related Proteins

**Figure 2:**
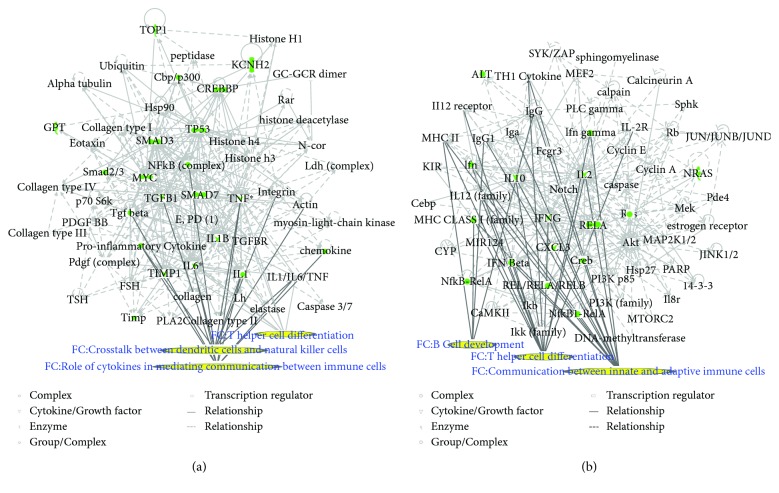
*Matrine* molecular networks. In each network, molecules are nodes, and a biological relationship between two nodes is represented by a line. Solid lines between molecules indicate a direct physical relationship between molecules, whereas dotted lines represent indirect functional relationships. Green symbols represent matrine-related proteins. Yellow symbols indicate the functional characteristics of the networks.

Two matrine molecular networks were established based on matrine-related proteins. As shown in Figure 2(a), some molecules in the network were linked to specific functions, including T helper (Th) cell differentiation, cytokine communication between immune cells, and crosstalk between dendritic cells and natural killer cells.  Figure 2(b) describes other specific functions. IPA data show that overall functions of both networks included cell-to-cell signaling and interaction, inflammatory responses, cellular growth and proliferation, and free radical scavenging. Thus, matrine may assist with immune regulation and anti-inflammatory responses.

### 3.3. Networks Associated with CRC-Related Genes

**Figure 3:**
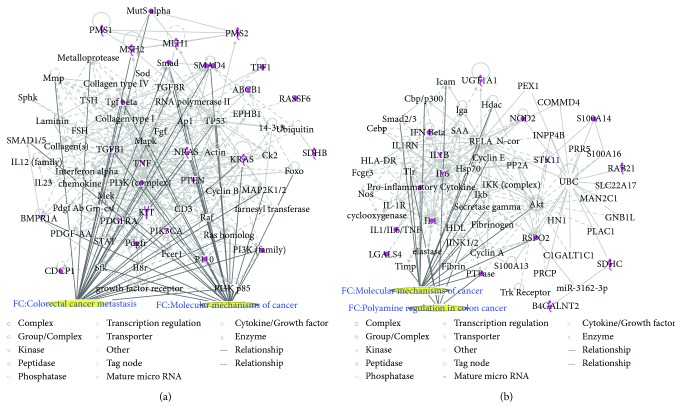
CRC molecular networks. In each network, molecules are nodes, and a biological relationship between two nodes is represented by a line. Solid lines between molecules indicate a direct physical relationship between molecules, whereas dotted lines represent indirect functional relationships. Purple symbols represent CRC-related genes. Yellow symbols indicate the functional characteristics of the networks.

Two CRC networks were established based on CRC-related genes.  Figure 3(a) shows that molecules were linked to specific functions, such as CRC metastasis and the molecular mechanisms of cancer. Additionally, polyamine regulation in colon cancer was a functional characteristic of the CRC networks (Figure 3(b)). Two CRC networks were associated with inflammatory and gastrointestinal diseases and organismal injury and abnormalities. This reflects the pathogenesis of CRC.

### 3.4. Merging of the Matrine and CRC Networks

**Figure 4:**
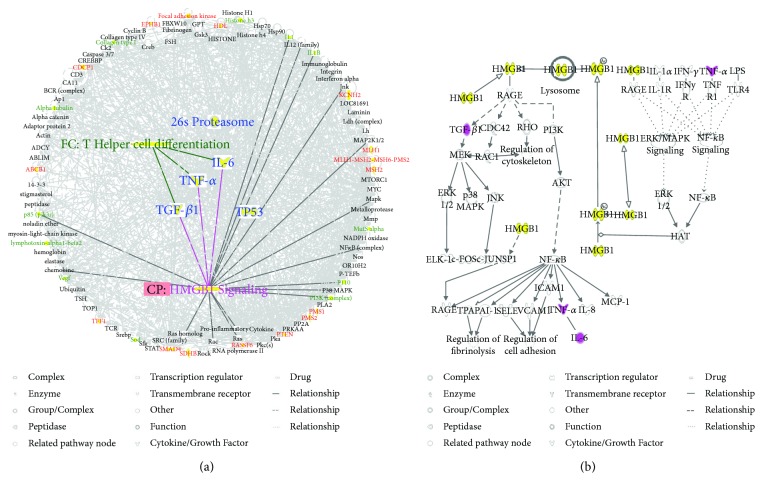
Common highly linked molecules and their most significantly related signaling pathways and functions in the “matrine-CRC” merged network. In the network, molecules are nodes, and biological relationships between two nodes are represented by lines. Solid lines between molecules show a direct physical relationship, whereas dotted lines show indirect functional relationships. (a) The five blue molecules in the center of the network represent common highly linked molecules. Red molecules represent CRC-related genes, and green molecules represent matrine-related proteins in the surrounding area of the network. In the Ingenuity Knowledge Database, “CP” is an abbreviation for “canonical pathway,” which represents a signaling pathway related to the highly linked molecules. “FC” is an abbreviation for “functional characteristic,” which represents functions related to the highly linked molecules. (b) Purple symbols represent highly linked molecules, and yellow symbols represent the molecules in the HMGB1 signaling pathway.

To predict targets of matrine intervention in CRC, matrine and CRC networks were compared, network dimensions were reduced, and the networks were merged with the IPA. As shown in Figure 4(a), five molecules, including IL-6, 26S proteasome, TNF-*α*, TGF-*β*1, and TP53, were identified as common linked molecules relevant to both matrine and CRC networks. According to the Ingenuity Knowledge Database, HMGB1 signaling was the most significantly related pathway for IL-6, TNF-*α*, and TGF-*β*1, and Th cell differentiation was the most significantly relevant function for these proteins. IL-6, TNF-*α*, and TGF-*β*1 were involved in HMGB1 signaling (Figure 4(b)). Thus, these five highly linked molecules and HMGB1 signaling were likely associated with the mechanism of matrine for treating CRC, and they may be potential target proteins of matrine.

### 3.5. Inhibitory Effect of Matrine on CRC Growth

Tumor number, weight, and size for each rat were recorded to evaluate antitumor effects of matrine.  Table 1 shows that compared to the model group, tumors in matrine-treated groups decreased. Additionally, tumor number, weight, and size in matrine-treated groups were fewer, and the HM group had the best outcome.

### 3.6. Effect of Matrine on Expression of Predicted Target Proteins

**Figure 5:**
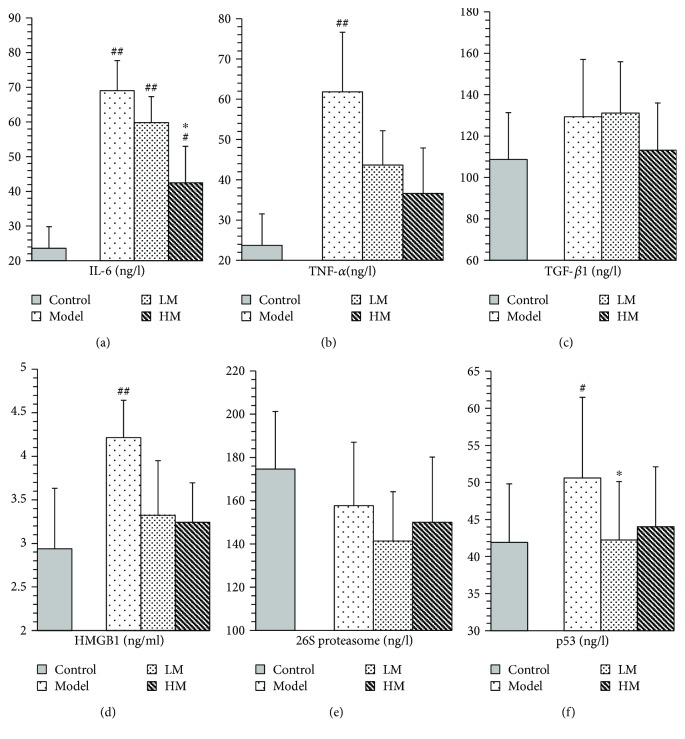
Expression of predicted target proteins in control, model and matrine-treated groups. Model, LM, and HM versus control, respectively: ^#^ *P* < 0.05 and ^##^ *P* < 0.01. LM and HM versus model, respectively: ^∗^ *P* < 0.05.

IL-6, TNF-*α*, HMGB1, and p53 were increased in the model group compared to the controls.  Figure 5 shows that after matrine treatment, IL-6 and HMGB1 in the HM group decreased, and p53 in the LM group decreased. TNF-*α* decreased in both matrine-treated groups. The decrease in TNF-*α* in the HM group was more significant than that in the LM group. No significant differences were found among groups with respect to 26S proteasome and TGF-*β*1.

## 4. Discussion

CRC is a leading cause of cancer-related deaths worldwide, and studies suggest that matrine may have antitumor effects and could have potential for treating CRC. However, how this occurs is not clear. A network pharmacology approach to understand mechanistic aspects of drugs may offer novel approaches for studying new compounds. We studied the effect of matrine on rats with CRC using this network pharmacology approach, and we observed that matrine significantly suppressed CRC growth; this was associated with dysregulation of specific proteins (IL-6, TNF-*α*, HMGB1, and p53) and a corresponding pathway (HMGB1 signaling) and a function (Th cell differentiation). To our knowledge, this study is the first report about the anti-CRC mechanism of matrine using network pharmacology.

IL-6 is mainly secreted by T cells and macrophages to stimulate an immune response and cause inflammation. IL-6 is critical for tumor microenvironment regulation and may be a key regulator during colorectal tumorigenesis via regulation of tumor-promoting inflammation [16–18]. Patients with advanced/metastatic cancer had high IL-6 [19, 20], and IL-6 expression was significantly elevated in CRC tissues compared to noncancerous tissues and was associated with invasiveness and lymph node metastasis [21]. Anti-IL-6 therapy was initially developed for treating autoimmune diseases, but the role of IL-6 in chronic inflammation suggests that IL-6 blockade may be feasible for cancer treatment [22, 23]. TNF-*α* is a cell signaling protein involved in systemic inflammation produced chiefly by activated macrophages. TNF-*α* plays a pivotal role in malignant cellular proliferation, angiogenesis, tissue invasion, and metastasis in CRC [24]. A previous study demonstrated that serum TNF-*α* may contribute to CRC susceptibility, and anti-TNF therapy was considered for CRC treatment [25]. In this study, matrine reduced elevated IL-6 and TNF-*α* in CRC, which was consistent with previous studies. TGF-*β*1 is a protein secreted by most immune cells, and it contributes to immune system control and performs cellular functions, including control of cell growth, proliferation, differentiation, and apoptosis [26]. A study showed that TGF-*β*1 promoted CRC immune escape [27]. TGF-*β*1 was increased in peripheral blood of CRC patients and may be associated with tumor size and location [28, 29]. Matrine did not affect TGF-*β*1 expression.

IL-6, TNF-*α*, and TGF-*β*1 are involved in HMGB1 signaling. HMGB1 is secreted by immune cells such as macrophages, monocytes, and dendritic cells, and it acts as a cytokine mediator of inflammation [30–33]. HMGB1 signals through the receptor for advanced glycation end-products (RAGE), a multiligand receptor of the immunoglobulin superfamily. Cell activation by HMGB1 causes release of proinflammatory cytokines such as IL-6, TNF-*α*, and TGF-*β*1 [34]. HMGB1 is key to cancer development, progression, and metastasis because it activates cancer cells, enhances tumor angiogenesis, and suppresses host anticancer immunity [35]. HMGB1 targeting has been identified as a potential therapeutic strategy against cancer development, progression, and in particular, metastasis [36]. We found that along with decreased IL-6 and TNF-*α*, matrine inhibited increases in HMGB1 in CRC, suggesting that HMGB1 and HMGB1 signaling may be relevant targets for CRC treatment.

p53 is a protein encoded by the TP53 gene, which is the most frequently mutated gene in human cancers and is a key to preventing cancer formation [37, 38]. TP53 was thought to be a potential predictive biomarker for CRC development and it has been used in the targeted therapy of CRC [39–41]. Matrine decreased expression of p53 in CRC, suggesting that targeting p53 might explain how matrine affects CRC. Proteasomes are critical to the function of the adaptive immune system by regulating expression of proinflammatory cytokine TNF-*α* [42]. Increased proteasome was correlated with autoimmune disease activity [43]. Proteasome inhibitors were effective against tumors in cell culture, inducing apoptosis by disrupting regulated degradation of progrowth cell cycle proteins [44]. Targeting the proteasome may thus be promising for treating CRC [45, 46]. Our data suggest that targeting the 26S proteasome may explain how matrine affects CRC.

## 5. Conclusion

Inhibition of HMGB1 signaling characterized by abnormal expression of specific proteins (IL-6, TNF-*α*, and HMGB1) relevant to Th cell differentiation was likely the underlying mechanism of CRC treatment by matrine. This finding might facilitate the identification of new targets for CRC treatment as well as offer information for novel targets and purported mechanisms for Chinese herbal medicine.

## Figures and Tables

**Table 1:** CRC in different groups (mean ± SD).

Groups (*n*)	Incidence (%)	Number	Weight (g)	Size (cm^3^)
Control (8)	0	0	0	0
Model (8)	100	2.63 ± 0.74	0.89 ± 0.86	1.09 ± 0.65
LM (8)	87.5	2.25 ± 0.71	0.17 ± 0.21^∗^	0.45 ± 0.50^∗^
HM (8)	75.0	1.50 ± 0.76^∗∗^	0.15 ± 0.17^∗^	0.28 ± 0.27^∗∗^

Note: *matrine*-administered groups compared to the model group. ^∗^ *P* < 0.05, ^∗∗^ *P* < 0.01.

## Supplementary Materials

Supplementary Materials Table S1: Matrine-related proteins. Table S2: CRC-related genes.
