# Supplementary material for: Matrine Ameliorates Colorectal Cancer in Rats via Inhibition of HMGB1 Signaling and Downregulation of IL-6, TNF-α, and HMGB1
Source: J Immunol Res. 2018 Jan 10;2018:5408324. doi: 10.1155/2018/5408324 (PMC5818890; doi:10.1155/2018/5408324)
Supplement: Supplementary Materials — Table S1: Matrine-related proteins. Table S2: CRC-related genes. [file 5408324.f1.docx]

**Supplementary materials**

**Table** **S1. Matrine-related proteins**

| **N** | **Symbol** | **Entrez Name** | **Location** |
| --- | --- | --- | --- |
| 1 | CREBBP | CREB binding protein | Nucleus |
| 2 | CXCL3 | chemokine (C-X-C motif) ligand 3 | Extracellular Space |
| 3 | GPT | glutamic-pyruvate transaminase (alanine aminotransferase) | Cytoplasm |
| 4 | IFNG | interferon, gamma | Extracellular Space |
| 5 | IL10 | interleukin 10 | Extracellular Space |
| 6 | IL1B | interleukin 1, beta | Extracellular Space |
| 7 | IL2 | interleukin 2 | Extracellular Space |
| 8 | IL-6 | interleukin 6 | Extracellular Space |
| 9 | KCNH2 | potassium channel, voltage gated eag related subfamily H, member 2 | Plasma Membrane |
| 10 | MYC | v-myc avian myelocytomatosis viral oncogene homolog | Nucleus |
| 11 | NRAS | neuroblastoma RAS viral (v-ras) oncogene homolog | Plasma Membrane |
| 12 | RELA | v-rel avian reticuloendotheliosis viral oncogene homolog A | Nucleus |
| 13 | SMAD3 | SMAD family member 3 | Nucleus |
| 14 | SMAD7 | SMAD family member 7 | Nucleus |
| 15 | TGFB1 | transforming growth factor, beta 1 | Extracellular Space |
| 16 | TIMP1 | TIMP metallopeptidase inhibitor 1 | Extracellular Space |
| 17 | TNF | tumor necrosis factor | Extracellular Space |
| 18 | TOP1 | topoisomerase (DNA) I | Nucleus |
| 19 | TP53 | tumor protein p53 | Nucleus |

**Table S2. CRC-related genes**

| **N** | **Symbol** | **Entrez Name** | **Location** |
| --- | --- | --- | --- |
| 1 | ABCB1 | ATP-binding cassette, sub-family B (MDR/TAP), member 1 | Plasma Membrane |
| 2 | B4GALNT2 | beta-1,4-N-acetyl-galactosaminyl transferase 2 | Cytoplasm |
| 3 | BMPR1A | bone morphogenetic protein receptor, type IA | Plasma Membrane |
| 4 | CDCP1 | CUB domain containing protein 1 | Plasma Membrane |
| 5 | CDH1 | cadherin 1, type 1, E-cadherin (epithelial) | Plasma Membrane |
| 6 | EPHB1 | EPH receptor B1 | Plasma Membrane |
| 7 | IL1B | interleukin 1, beta | Extracellular Space |
| 8 | IL1RN | interleukin 1 receptor antagonist | Extracellular Space |
| 9 | IL-6 | interleukin 6 | Extracellular Space |
| 10 | KIT | v-kit Hardy-Zuckerman 4 feline sarcoma viral oncogene homolog | Plasma Membrane |
| 11 | KRAS | Kirsten rat sarcoma viral oncogene homolog | Cytoplasm |
| 12 | LGALS4 | lectin, galactoside-binding, soluble, 4 | Extracellular Space |
| 13 | MLH1 | mutL homolog 1 | Nucleus |
| 14 | MSH2 | mutS homolog 2 | Nucleus |
| 15 | NOD2 | nucleotide-binding oligomerization domain containing 2 | Cytoplasm |
| 16 | NRAS | neuroblastoma RAS viral (v-ras) oncogene homolog | Plasma Membrane |
| 17 | PDGFRA | platelet-derived growth factor receptor, alpha polypeptide | Plasma Membrane |
| 18 | PIK3CA | phosphatidylinositol-4,5-bisphosphate 3-kinase, catalytic subunit alpha | Cytoplasm |
| 19 | PMS1 | PMS1 postmeiotic segregation increased 1 (S. cerevisiae) | Nucleus |
| 20 | PMS2 | PMS2 postmeiotic segregation increased 2 (S. cerevisiae) | Nucleus |
| 21 | PRAC2 | prostate cancer susceptibility candidate 2 | Other |
| 22 | PTEN | phosphatase and tensin homolog | Cytoplasm |
| 23 | RAB21 | RAB21, member RAS oncogene family | Cytoplasm |
| 24 | RASSF6 | Ras association (RalGDS/AF-6) domain family member 6 | Other |
| 25 | RSPO2 | R-spondin 2 | Extracellular Space |
| 26 | S100A14 | S100 calcium binding protein A14 | Cytoplasm |
| 27 | SDHB | succinate dehydrogenase complex, subunit B, iron sulfur (Ip) | Cytoplasm |
| 28 | SDHC | succinate dehydrogenase complex, subunit C, integral membrane protein, 15kDa | Cytoplasm |
| 29 | SMAD4 | SMAD family member 4 | Nucleus |
| 30 | STK11 | serine/threonine kinase 11 | Cytoplasm |
| 31 | TFF1 | trefoil factor 1 | Extracellular Space |
| 32 | TGFB1 | transforming growth factor, beta 1 | Extracellular Space |
| 33 | TNF | tumor necrosis factor | Extracellular Space |
| 34 | TP53 | tumor protein p53 | Nucleus |
| 35 | UGT1A1 | UDP glucuronosyltransferase 1 family, polypeptide A1 | Cytoplasm |
